# Development of Highly Hygienic Textile by Coating with Encapsulated Ginseng Oil

**DOI:** 10.3390/polym15224352

**Published:** 2023-11-08

**Authors:** Sujin Ryu, Jaeyun Shim

**Affiliations:** Advanced Textile R&D Department, Research Institute of Convergence Technology, Korea Institute of Industrial Technology (KITECH), 143 Hanggaulro, Sangnok-gu, Ansan-si 15588, Gyeonggi-do, Republic of Korea

**Keywords:** ginseng microcapsule, encapsulation, hygienic textile, multifunctional fabric

## Abstract

There is a growing demand for the development of functional textile sanitary products to protect the human body from viruses, bacteria, and other harmful external substances. However, common processing methods for textile functionalization result in poor durability or have a highly limited material scope. A solution for this is the encapsulation of the functional material to provide stable protection and controlled release to reveal functionality in the fabric. However, many chemicals used for such purposes can cause problems for both human beings and the environment; therefore, attention is being shifted to natural products such as essential oils and seed oils. In this study, we used in situ polymerization to encapsulate ginseng oil, which has antibacterial, deodorizing, moisturizing, and antioxidant functions, as the core material of the microcapsules. The manufactured microcapsules were spherical with smooth surfaces, had an average size of 3.98 um, and exhibited excellent thermal stability. Processing the synthesized microcapsules into nylon/polyurethane fabric resulted in excellent functionalities, with the treated fabric exhibiting a 99.9% antibacterial activity against *Staphylococcus aureus* and *Klebsiella pneumoniae* and a 99% deodorizing effect. Therefore, the developed method is expected to show great potential for the production of highly hygienic textiles for use in various industries.

## 1. Introduction

The functionalization of textile products for use as personal protective equipment (PPE), filters, sensors, clothing, interior materials, and so on is being actively pursued in various industries, such as hygiene, interior design, and medical care, using fiber as the substrate [1,2,3]. In particular, owing to the recent COVID-19 pandemic, the development of PPE products is needed to protect the human body from external bacteria or viruses and harmful substances. The WHO has also announced recommendations regarding PPE to prevent the spread of infectious diseases, resulting in the increased use of facial masks and other PPE [4,5]. While physical blocking is sufficient to guard against the coronavirus, which is transmitted through droplets, functional products are required to directly remove bacteria and viruses that are transmitted through the air or skin.

Functionalities such as antibacterial properties and the ability to adsorb and remove harmful substances can be imparted to textile products via various methods, including directly coating fibers with functional substances, mixing functional substances with fibers and spinning them, and encapsulating functional substances and attaching them to fibers [6]. The direct application of functional substances to fibers is the simplest and most widely used method, but, owing to the low durability of the resulting fabric, volatile substances cannot be used; furthermore, the functional substances can make the textile products stiff or degrade their feel and appearance [7,8]. Mixing functional materials with fibers and spinning them results in higher durability; however, the scope of the materials that are compatible with fiber polymers is highly limited [9]. Encapsulation is a method in which functional materials can be surrounded by wall material via an appropriate polymerization method, depending on the characteristics of the core material [10,11,12,13]. This method can improve durability by effectively protecting the core material; furthermore, the wall thickness can be adjusted to appropriately control the release of the encapsulated material [14].

Encapsulation methods are largely classified as spray drying, coacervation, and in situ polymerization and can be chosen depending on the core material [15]. Spray drying involves the drying of liquid substances into a powder form by exposing them to hot air under applied pressure. This method facilitates easy mass production but cannot be applied when the wall material has high hardness, which prevents the product from exhibiting controlled release; therefore, it is mainly used in the food industry [16,17]. Coacervation is based on the principle that a single-substance polymer solution under thermodynamic equilibrium separates into two separate layers, the dispersion and succession phases, upon the addition of other substances. This method is sensitive to temperature and pH changes, making it difficult to use for mass production; therefore, it is used In industries such as pharmaceuticals and cosmetics [18,19]. In situ polymerization is one of the most commercialized methods. It involves the emulsion of two immiscible materials using the phase of one material as a solvent, followed by the wrapping of the emulsion with a prepolymer resin to encapsulate it. This method results in excellent durability and can be used with phase-change materials and aromatic materials [20,21,22].

Chemicals with antibacterial activity have previously been applied in the form of capsules, owing to their low cost; however, such chemicals can cause alternate problems to the human body and the environment. Therefore, research is actively underway to replace them with natural substances, such as essential oils [23,24,25,26]; however, depending on the type and the amount of aromatic essential oil, high functionality may be difficult to achieve. Therefore, some studies have used herbs, oils, and medicinal plants to process textile products [27,28,29,30]. Ginseng oil contains a large number of pharmacological ingredients and has been reported to have antibacterial, deodorizing, anti-inflammatory, and antioxidant effects as well as skin-moisturizing, wrinkle-improving, and atopic relief effects [31,32,33]. Ginseng oil is not a single molecule but a mixture of several compounds, such as alkaloids, phenols, terpenes, proteins, and fatty acids [31]. Among them, alkaloids interfere with bacterial DNA replication, and phenols and terpenes destroy bacterial cell membranes and prevent bacterial growth [33]. Herbs, including ginseng, can adsorb and neutralize odors, neutralize odors with the scent of volatile oils, or remove odors by volatilizing ammonia molecules together [30]. As a secondary factor, the antibacterial activity of ginseng oil can eliminate bacteria and mold that cause odor. However, despite many reported pharmacological effects, ginseng oil has never been used in textile finishing for the purpose of imparting functionality because of cost. Compared to the entire-area coating method, the microcapsule coating method may be more complicated. However, since it uses a stirrer in the synthesis process and a padder in the coating process, it can be produced using similar processes and equipment as the entire-area coating method. As a result, when considering the cost aspect, excellent durability can be achieved by using a small amount. Therefore, we expect that encapsulating ginseng oil and processing it into fibers can afford functionalities, such as antibacterial and deodorizing properties. At this time, an MF prepolymer was synthesized by using melamine and formaldehyde as the wall material of the microcapsule, and this was used to construct a capsule wall that was strong against external heat or pressure. This allowed the ginseng oil in the capsule to be released continuously, rather than releasing the internal substances all at once. The wall of the microcapsule attached to the fabric gradually releases ginseng oil due to external factors, and as the ingredients in the ginseng oil volatilize, they can remove odor or inhibit the growth of bacteria.

**Figure 1:**
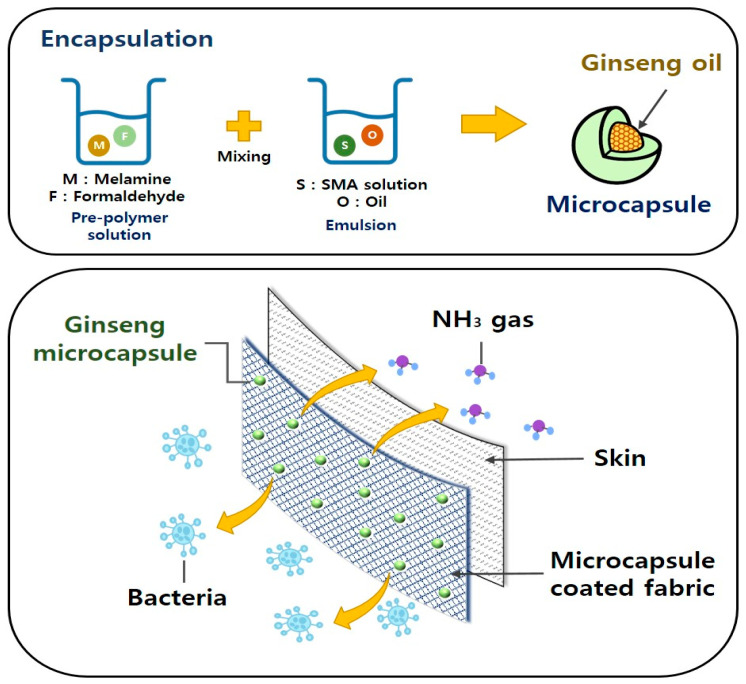
Scheme of ginseng oil encapsulation and fabrication of hygienic textile via microcapsule coating.

In this study, we report an encapsulation route for ginseng oil via in situ interfacial polymerization. The resulting microcapsules were applied to fabric to impart it with antibacterial and deodorizing properties (Figure 1). The antibacterial activity was tested against two bacterial strains, *Staphylococcus aureus* and *Klebsiella pneumoniae*, and deodorizing performance was tested by measuring the adsorption of ammonia gas. Thus, ginseng microcapsules were successfully used to fabricate highly hygienic fabric, highlighting their potential for application in various types of hygienic products based on the fabric.

## 2. Materials and Methods

### 2.1. Materials

The melamine monomer 2,4,6-triamino-1,3,5-triazine (sym-triaminotriazine; Sigma–Aldrich Co., Ltd., Steinheim am Albuch, Germany) and formaldehyde solution (37 wt%, Sigma–Aldrich Co., Ltd., Germany) were used as the wall materials for microcapsule synthesis. Super-critically extracted ginseng oil (Goldleben, Cheongju, Republic of Korea) and soybean oil (Sigma–Aldrich Co., Ltd., Germany) were used as the core materials. The copolymer stylene maleic anhydride (SMA; scirptset 520, Solutia Inc., St. Louis, MO, USA) was used as the emulsifier in the emulsification process. The synthesized microcapsules were attached to a 240-g/yd nylon/polyurethane (PU) (77/23) fabric using a fiber binder (7PGN, HD Resin Inc., Seongnam, Republic of Korea). All materials were used as received without further purification.

### 2.2. Fabrication and Coating of Ginseng Microcapsules

**Figure 2:**
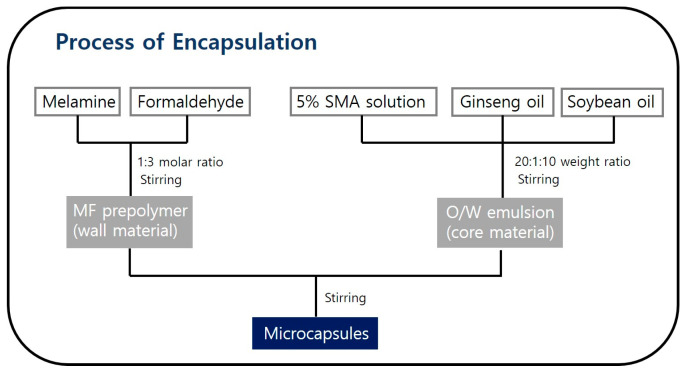
Schematic diagram of the process of encapsulation.

A melamine–formaldehyde prepolymer (MF) was synthesized as the wall material by using the common method of mixing melamine and formaldehyde at a molar ratio of 1:3 and stirring at 500 rpm at 60 °C for 15 min. Since melamine is composed of a triazine ring, it can react with approximately 3 formaldehydes per molecule. The stirring speed and time are sufficient to mix evenly, but a temperature of 80 °C or lower is recommended because ginseng oil begins to volatilize around 80 °C. The polymerization process of the MF prepolymer is shown in Figure S1. For the core material, an aqueous solution of 5 wt% SMA was mixed with ginseng seed oil and soybean oil at a weight ratio of 20:1:10, and the mixture was stirred at 500 rpm for 10 min to prepare an aqueous emulsion. The synthesized prepolymer was then added to the ginseng oil emulsion and stirred at 1500 rpm at 70 °C for 1 h using a homogenizer (T-25, Ika, Co., Ltd., Staufen, Germany), followed by stirring at 1000 rpm for 5 h at room temperature of 20 °C. Upon cooling, the desired ginseng microcapsules were obtained. An overall schematic diagram of the encapsulation process is shown in Figure 2.

The nylon/PU fabric was immersed in the 1.0 wt% microcapsule solution and fixed by curing with a resin binder. Then, a padder (DL-2005, Daelim starlet, Republic of Korea) was employed at 0.5 bar pressure and a rate of 1 m/min to achieve a 60% wet pick-up.

### 2.3. Characterization

The surface characteristics of the synthesized ginseng microcapsules, nylon/PU substrate, microcapsule-coated fabric, and microcapsule-coated fabric washed repeatedly 5 times were observed using a field emission scanning electron microscope (FE-SEM; SU8010, Hitachi, Tokyo, Japan). Fourier-transform infrared (FT-IR) spectroscopy was conducted on the ginseng oil and the synthesized ginseng microcapsules using an infrared spectrometer (Spectrum Two FT-IR Spectrometer, Perkin Elmer, Waltham, MA, USA) to confirm synthesis. The average particle size of the synthesized ginseng microcapsules was analyzed using a particle size analyzer (Mastersizer 2000, Malvern, Republic of Korea). The thermal stability of ginseng oil and the ginseng microcapsules was evaluated using thermogravimetric analysis (TGA; Q600, TA Instruments, New Castle, DE, USA) over a temperature range of 30–600 °C at a rate of 10 °C/min. A total of 0.2 g of the ginseng microcapsules was dispersed in 100 mL of distilled water, and the absorbance was measured using a UV-Vis spectrophotometer (Shimadzu, Kyoto, Japan) at 275 nm, which is the maximum absorption wavelength of ginseng oil. The release rate of the ginseng oil from the microcapsules was estimated using Equation (1).

(1) $$Release\;rate\;(\%)=\frac{A_{f}-A_{i}}{A_{i}}\times 100$$

### 2.4. Functionality Evaluation

The antibacterial activity of the microcapsule-treated fabrics was evaluated based on the ISO 20645:2006 [34]. *Staphylococcus aureus* (ATCC 6538) and *Klebsiella pneumoniae* (ATCC 4352) were used as the test bacteria strains. The bacterial reduction rate (%), or the bacteriostatic rate, is the relative reduction rate of the number of viable bacteria in each sample. It was calculated for the control and test samples after 18 h of incubation using the bacterial count as follows:

(2) $$Bacterial\;reduction\;rate\left(\%\right)=\frac{M_{b}-M_{c}}{M_{b}}\times 100,$$

where M_b_ and M_c_ are the numbers of microbes in the blank and test specimens, respectively, after incubation for 18 h.

The deodorizing properties of the nylon/PU substrate and the ginseng microcapsule-treated fabrics were evaluated using a gas detection tube with which the extinction rate of ammonia gas was measured. After placing a 10 cm × 10 cm section of the fabric and 500 µg/mL ammonia gas in a 1000 mL container, the extinction rate of the ammonia gas was measured for 30, 60, 90, and 120 min. Then, the ammonia gas concentration was measured, and the deodorization rate was calculated as follows:

(3) $$Deodorization\;rate\left(\%\right)=\frac{A-B}{A}\times 100,$$

where A and B are the ammonia gas concentrations in sealed jars containing the blank and the sample, respectively.

## 3. Results and Discussion

### 3.1. Fabrication of Ginseng Microcapsules

**Figure 3:**
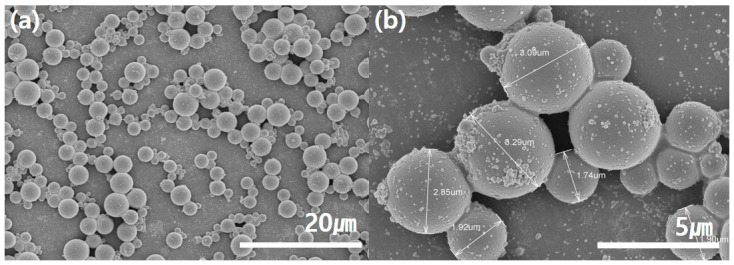
SEM images of fabricated ginseng microcapsules magnified (**a**) ×500 and (**b**) ×2000.

Microcapsules containing ginseng oil were prepared via in situ polymerization. In situ polymerization requires the interfacial polymerization of two immiscible substances using the phase of one substance as a solvent and then wrapping the core material with a prepolymer resin. Therefore, first, an MF prepolymer was prepared by mixing the melamine and formaldehyde solutions. Then, interfacial polymerization was conducted using an oil phase (ginseng and soybean oils) and a water phase as the emulsifier (SMA solution). The polymerized emulsion was then encapsulated by stirring the MF prepolymer. SEM images of the synthesized ginseng microcapsules are shown in Figure 3. The capsules exhibited smooth surfaces and excellent durability; these results are in agreement with those of previous studies, which state that using a resin series such as urea–formaldehyde or MF as the prepolymer can produce smooth, durable capsules [35,36]. Furthermore, from the SEM images, the microcapsules were found to be stable, spherical, and approximately 3 um in size.

### 3.2. Characteristics of the Fabricated Ginseng Microcapsules

**Figure 4:**
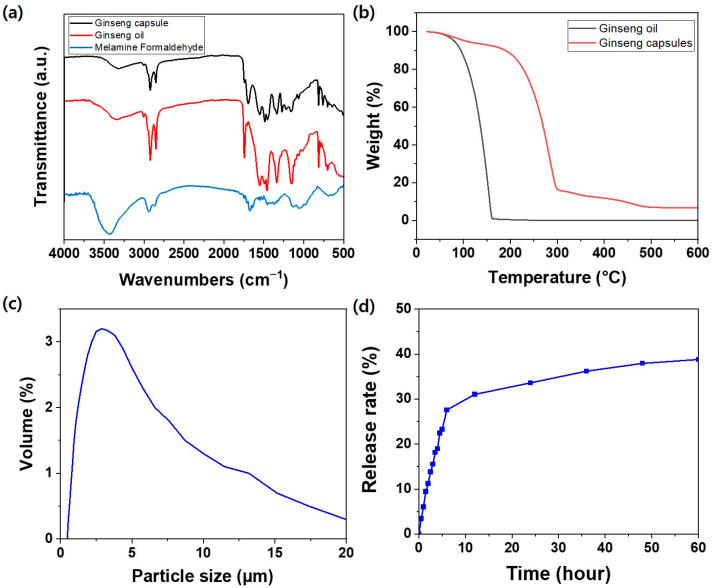
(**a**) FT-IR spectra of ginseng microcapsules (black), ginseng oil (red), and melamine–formaldehyde prepolymer (blue). (**b**) TGA curves of ginseng oil (black) and ginseng microcapsules (red). (**c**) Particle size distribution of ginseng microcapsules. (**d**) Release property of ginseng microcapsules.

The fabricated ginseng microcapsules were characterized by conducting FT-IR spectroscopy on the microcapsules, MF prepolymer, and ginseng oil (Figure 4a). In the MF prepolymer spectrum, characteristic peaks were observed at 3432 and 1676 cm^−1^, corresponding to the NH stretching vibration characteristic of melamine and the C–O bond peak of the amide group caused by the cross-linking of MF, respectively [37,38]. As the same peaks also appeared in the FT-IR spectrum of the microcapsules, it was confirmed that MF was a component of the microcapsules. Meanwhile, ginseng oil contains not only ginsenosides but also various components such as aromatic compounds, carbohydrates, and proteins. Therefore, in the ginseng oil spectrum, strong peaks appeared at 2923 cm^−1^ (C–H stretching), 2852 cm^−1^ (stretching vibration of the –CH_2_– groups), and 1742 cm^−1^ (stretching vibration of the C=O group in volatile oils and other carbonyl-containing compounds) [39]. Notably, oils are characterized by strong characteristic peaks in FT-IR spectra. Therefore, because the characteristic peaks of ginseng oil appeared at 2923 cm^−1^ and 2852 cm^−1^ and are similar to those of the MF prepolymer characteristic peaks, the appearance of strong peaks in the spectrum of the synthesized microcapsules indicates the presence of ginseng oil. For the ginseng capsule, which is encapsulated with an MF prepolymer as the wall material and contains ginseng oil as the core material, the peak at 2923 cm^−1^ is due to C–H stretching, the peak at 2852 cm^−1^ is due to the stretching vibration of the –CH_2_– groups, and the peak at 1650 cm^−1^ is due to the C–O bond. As a result, it can be confirmed that the synthesis was successful because the characteristic peaks of both the MF prepolymer and the ginseng oil appear in the capsule.

Oils are volatile substances, and volatilization occurs even at room temperature and increases upon applying heat, resulting in significant weight loss. Therefore, when used in fabric processing, it is necessary to protect oils from volatilization by microencapsulating them. The change in weight of the ginseng oil and the synthesized microcapsules with increasing temperature was measured using TGA (Figure 4b). Ginseng oil began undergoing rapid weight loss starting at 80 °C, and the entire amount was volatilized before 200 °C. In contrast, the ginseng microcapsules, despite losing some weight due to the evaporation of moisture and the destruction of the microcapsule structure, retained more than 90% of their weight upon increasing the temperature to 200 °C. Therefore, the microcapsules can be considered to have thermal stability.

The sizes of the microcapsules are important factors in determining their functionality. If the microcapsules are too small, it is difficult to achieve sufficient functionality because the amount of internal material carried will be insufficient. Meanwhile, if the microcapsules are too large, they may block the pores between the fibers, which can reduce air permeability and affect the surface feel of the fabric. Figure 4c shows the particle size distribution of the synthesized ginseng microcapsules. Similar to the size shown in the SEM images (Figure 3), most particles were approximately 3 um in size. During the dispersion of the microcapsules in the particle size measurement process, some of the microcapsules aggregated; these aggregates were considered as individual particles with sizes greater than 5 um. Regardless, the average particle size was 3.98 um. Therefore, the microcapsules were of an appropriate size compared to the fiber diameter of the nylon/PU fabric used in this study. Figure 4d shows the release property of the ginseng microcapsules from the time of fabrication until 60 h had passed. It tended to be released rapidly until 10 h had elapsed, but after that, sustained release was confirmed to continue until 60 h had elapsed. In the case of processing for disposable textiles, excessive ginseng oil can be released by minimizing the ratio of MF resin, which is the wall material of the capsule. On the other hand, in the case of reusable textiles, the slow release of ginseng oil can be controlled by increasing the ratio of MF resin.

### 3.3. Surface Morphology of Ginseng Microcapsule-Coated Fabric

**Figure 5:**
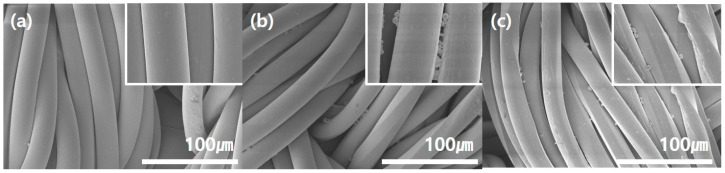
SEM images of the surface morphologies of the (**a**) nylon/PU substrate, (**b**) ginseng microcapsule-coated fabric, and (**c**) ginseng microcapsule-coated fabric after repeated washing 5 times.

To understand the effect of microcapsule coating on the surface of the fabric, the surface morphologies of the nylon/PU substrate (Figure 5a) and the fabric coated with the ginseng microcapsules at a concentration of 1% (Figure 5b) were compared. The untreated nylon/PU substrate exhibited a smooth surface, while the microcapsule-treated fabric had microcapsules attached between the fibers. In the case of applying functional materials to the entire fabric, which is the most commonly used method, the viscosity of the solution may have a negative effect on the appearance of the fabric or may block pores depending on the type of material. In fact, in this study, in the case of the microcapsule-treated fabric, the thickness of the substrate before coating was 0.257 mm, and after coating, it was 0.258 mm, which is a very small difference. In addition, as can be seen in the SEM image, there was a small amount of coating between the fabrics, so there was no change in the roughness of the coating surface. Notably, from the enlarged SEM image, empty space could be observed between the microcapsules, indicating that the pores were not blocked; this is because the fiber diameter was ≈20 um, while the microcapsule size was ≈3 um, as derived from particle size analysis and the SEM images. Therefore, there were no significant effects on the air permeability and feel of the fabric. Furthermore, as shown in Figure S2, microcapsules were present in the fabric even at the lower concentrations of 0.1–0.8%, and the number of microcapsules attached to the fibers increased with increasing concentration. Therefore, a concentration of 0.1% is sufficient for fabricating a ginseng microcapsule-treated fabric; however, a concentration of 1% is preferable to achieve appropriate functionality. Figure 5c shows the surface morphologies of the ginseng microcapsule-coated fabric after repeated washing five times. As the binder was washed away, it appeared that the microcapsules that were fixed with the binder fell off slightly. However, since the microcapsules still remained in the fabric even after washing five times, the durability against the physical force of washing was confirmed.

### 3.4. Evaluation of Functionality

For textiles that come into direct contact with the skin, the ability to block external bacteria and quickly remove bacteria generated from the body is crucial from a hygiene perspective. Therefore, the antibacterial properties of the ginseng microcapsule-coated fabric were evaluated using methods described in previous studies [40,41] using two bacterial strains: *S. aureus* and *K. pneumoniae*. These Gram-negative bacteria are the causative agents of food poisoning and easily attach to underwear or pants. They can enter the human body through various points such as the mouth, nose, ears, and skin and can be transmitted via bedding, clothing, and the air; they are, therefore, classified according to the KS K ISO 20645:2004.

**Figure 6:**
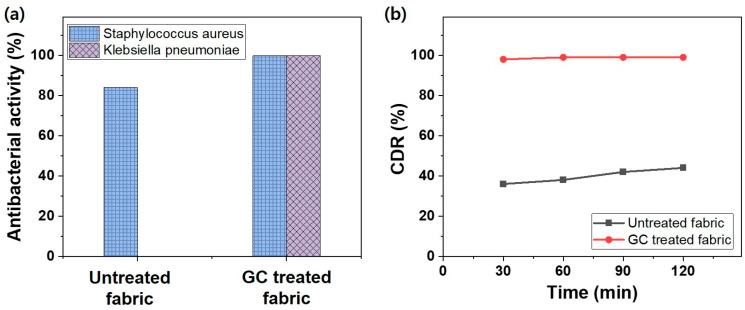
Functionality of the untreated and ginseng microcapsule (GC)-treated fabrics: (**a**) antibacterial and (**b**) deodorizing activities.

As shown in Figure 6a, the nylon/PU substrate exhibited an excellent 84.1% bacteriostatic reduction rate for *S. aureus* but a 0% reduction in *K. pneumoniae*; that is, no *K. pneumoniae* were removed. In contrast, the fabric coated with 1% ginseng microcapsules successfully removed 99.9% of both bacterial strains. The ginseng oil contained in the microcapsules applied to the nylon/PU fabric was continuously released to produce antibacterial action. In particular, alkaloids in ginseng oil interfere with bacterial DNA replication. Interestingly, as shown in Figure S3, while the bacteriostatic reduction rate for *S. aureus* slightly increased with increasing microcapsule concentration, even at concentrations lower than 1%, *K. pneumoniae* could not be removed when the microcapsule concentration was less than 0.8%. Therefore, it can be concluded that a minimum ginseng microcapsule concentration of 1% is required to release an appropriate amount of ginseng oil for bacteriostatic reduction.

Deodorizing activity can also be considered an important function of a fabric from a hygiene perspective. Sweat and other body secretions cause bad odors, and the volatilization of their ammonia component can have adverse effects on the human body, such as skin rashes [42]. Therefore, the deodorizing properties of the nylon/PU substrate and the fabric coated with the 1% ginseng microcapsules were evaluated by exposing them to ammonia gas and measuring the adsorption for up to 120 min (Figure 6b). In the case of the nylon/PU substrate, the rate by which the ammonia gas concentration decreased (CDR) was only 44% even after 120 min of exposure. In contrast, the ginseng microcapsule-coated fabric exhibited excellent adsorption properties, with a CDR value of more than 98% after only 30 min of exposure and an incredible 99.9% after 60 min. Ginseng can adsorb and neutralize odors, neutralize odors with the scent of volatile oils, or remove odors by volatilizing ammonia molecules together. As a secondary factor, the antibacterial activity of ginseng oil can eliminate bacteria and mold that cause odor. Thus, the encapsulation of ginseng oil and its coating of the fabric resulted in the successful functional expression of the antibacterial and deodorizing properties of ginseng oil in the fabric.

## 4. Conclusions

In this study, microcapsules containing ginseng oil were manufactured for use in functional textile processing using natural products that have a low burden on the environment and the human body. The synthesized microcapsules were characterized in terms of their surface morphology, thermal stability, and particle size. The microcapsules were then processed into fibers, and the expression of the functionality of the internal material in the fabric was confirmed.

The manufactured microcapsules were spherical and had smooth surfaces, and FT-IR spectroscopy confirmed the presence of ginseng oil at their cores. It also proved that microencapsulation provided excellent thermal stability. In addition, the synthesized microcapsules were of an appropriate size for textile processing, and it was confirmed that the synthesized capsules continuously released ginseng oil. The fabric was successfully coated with the microcapsules, and the surface to which the microcapsules were attached could be confirmed even after five repeated washings. The number of microcapsules was sufficient to provide functionality while not blocking the pores of the fabric. Upon testing, the fabric treated with the ginseng microcapsules successfully exhibited excellent antibacterial and deodorizing properties

We believe that the finishing method introduced in this study can contribute to the development of hygienic textile materials by facilitating the stable protection and appropriate release of functional substances. Furthermore, in the case of ginseng oil, additional moisturizing and antioxidant effects can also be expected, which can increase its application or scope in various textile fields, such as mask packs that come into direct contact with the skin and innerwear for patients with skin diseases.

## Data Availability

The data presented in this study are available on request from the corresponding author.

## Supplementary Materials

The following supporting information can be downloaded at: https://www.mdpi.com/article/10.3390/polym15224352/s1, Figure S1: Chemical reaction of the synthesis of melamine and formaldehyde. Figure S2: SEM images of the surface morphologies of the ginseng microcapsule-coated fabric according to the concentration of capsules: (a) 0.1 %, (b) 0.2%, (c) 0.3%, (d) 0.5%, and (e) 0.8%. Figure S3: Antibacterial activity of the ginseng microcapsule-coated fabric according to the concentration of capsules.
