# Supplementary material for: Development of Highly Hygienic Textile by Coating with Encapsulated Ginseng Oil
Source: Polymers (Basel). 2023 Nov 8;15(22):4352. doi: 10.3390/polym15224352 (PMC10674727; doi:10.3390/polym15224352)
Supplement: Supplementary file 1 [file polymers-15-04352-s001.zip › polymers-2697142-supplementary.pdf]

Supporting Information

Development of Highly Hygienic Textile by Coating Encapsulated Ginseng Oil

Sujin Ryu and Jaeyun Shim *

[1]Advanced Textile R&D Department, Research Institute of Convergence Technology, Korea Institute of Industrial Technology (KITECH), 143 Hanggaulro, Sangnok-gu, Ansan-si 15588, Gyeonggi-do, Republic of Korea

* Correspondence: sjaeyun@kitech.re.kr

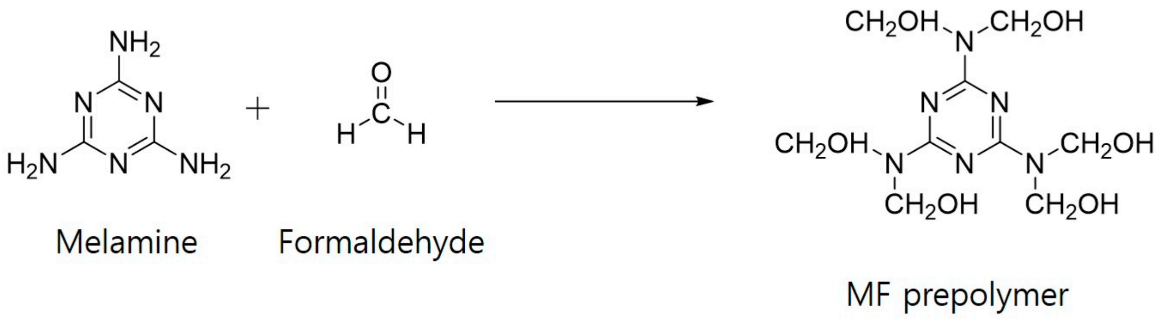

Figure S1. Chemical reaction of synthesis of melamine and formaldehyde.

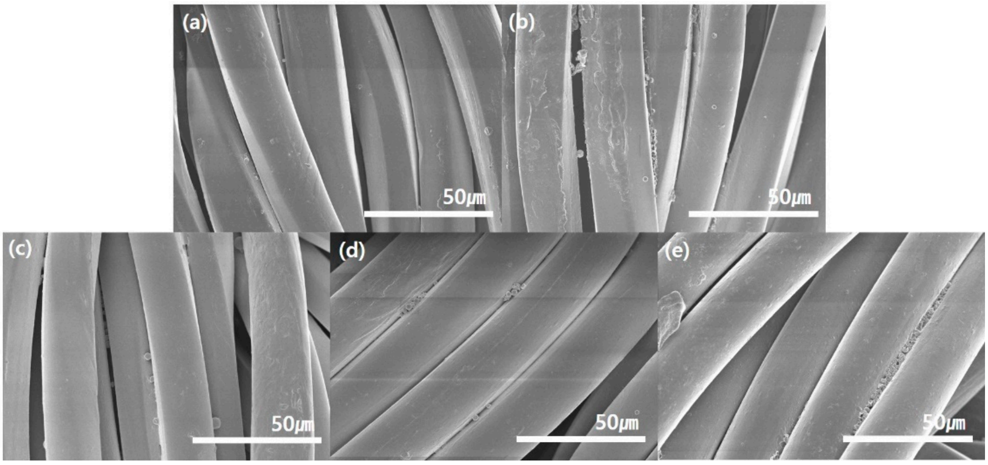

Figure S2. SEM images of the surface morphologies of the Ginseng microcapsules coated fabric according to concentration of capsules (a) 0.1 %, (b) 0.2%, (c) 0.3%, (d) 0.5% and (e) 0.8 %.

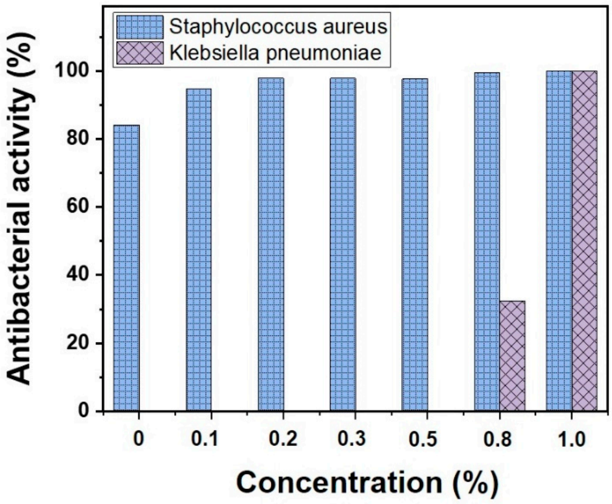

Figure S3. Antibacterial activity of the ginseng microcapsules coated fabrics according to concentration of capsules
